# Editorial: Physical activity and lifestyle sustainability: From childhood to old age

**DOI:** 10.3389/fpubh.2022.1097451

**Published:** 2023-01-06

**Authors:** Bojan Masanovic, Stevo Popovic, Juel Jarani, Radenko M. Matic

**Affiliations:** ^1^Faculty for Sport and Physical Education, University of Montenegro, Niksic, Montenegro; ^2^Western Balkan Sport Innovation Lab, Podgorica, Montenegro; ^3^Faculty of Movement Sciences, Sports University of Tirana, Tirana, Albania; ^4^Faculty of Sport and Physical Education, University of Novi Sad, Novi Sad, Serbia

**Keywords:** healthy lifestyle, exercise, children, adults, elderly, sedentary behavior

## Introduction

The industrial revolution from the second half of the previous century and the communication revolution, which has been especially noticeable in the last 20 years, caused a change in lifestyle and forced man to adapt to his inventions (1). This led to a change in the behavior of children, adults, and the elderly, and more and more negatively contributes to their development, their working ability, and their health condition (2). The physical inactivity of children during the period of intensive physical and cognitive development will cause their genetic potentials are not fully developed and they more easily create bad behavioral habits which can be the generator of many health problems in old age (3).

Facing the negative trends of the modern way of life (given that previous research clearly indicates that physical inactivity and sedentary behavior can lead to impaired health, i.e., that an active lifestyle can ensure a healthy life and promote wellbeing for all) this Research Topic was created to develop knowledge and an understanding of physical activity and lifestyle sustainability (from childhood to old age); and thus to help identify possible solutions and strategies that provide (4) help the upbringing of strong children, a (2) healthy transition from childhood, *via* adulthood, to old age, in the long term, and also (5) to solving problems of adults and elderly.

The purpose of this Research Topic was to build a collection of new knowledge about the positive impact of physical activity on the health and wellbeing of community members of all age groups (From Childhood to Old Age). There are 24 articles included in this Research Topic.

Most of the studies from this Research Topic (six of them) studied the impact of physical activity on physical fitness and health status (two of them are systematic reviews and studied the period from 2004 to 2020; two of them are longitudinal studies and follow the changes after 5 weeks and after 8 years) among young and elderly. All of them agree in the conclusion that any kind of additional physical activity (even when it is only housework or active transport) if it is used continuously and not occasionally, has beneficial effects on physical fitness and health status (Bai et al.; Xiao et al.; Lomsdal et al.; Huang et al.; Baj-Korpak et al.), also that people who are more physically active achieve better physical fitness, overall health and wellbeing from less active ones (TeraŽ et al.).

Furthermore, five cross-sectional studies were interested in evaluating the impact of the COVID-19 pandemic on the amount of physical activity and the lifestyle of students and the elderly. Based on the results of four of these five studies, it is known that there have been certain changes in the amount of physical activity and lifestyle, i.e., that sedentary behavior and passive activities increase both in the student population and in the elderly population, but that students were able to maintain the recommended amount of physical activity, and their physical activity declined to a lesser extent than in the elderly (Sekulic et al.; Kim et al.; Pišot et al.; Obradovic et al.). The fifth study is differently conceived, but extremely interesting, it describes the diet of Lithuanian and Croatian students in this difficult period and shows that those students who are more physically active took better care of proper nutrition, which means that care for one type of healthy way of life encourages care for others (Mieziene et al.).

The following four studies presented new study protocols, scales for testing, and national surveillance systems which are designed and validated to assess and follow-up physical activity, physical fitness, and health status of their population (7–8 years aged children, pregnant females, adults and older adults) (Mutare et al.; Zhu et al.; Zhang et al.; Jurak et al.).

Another important stream of work is reflected in the next four studies that analyzed the influence of sociodemographic factors on health behaviors, physical activities, physical fitness, and physical competence of children, adults, and the elderly. In each of the studies, there is evidence that sociodemographic factors (gross national income, income, and education) are positively correlated with behaviors, with the extent of physical activity and with physical performance of children and elderly, also that football players from less economically developed countries in running performance are below the values achieved by those who compete in the countries of the European Union (Rodriguez-Rodriguez et al.; Liu Z. et al.; Santibañez-Gutierrez et al.; Goranovic et al.).

The last five studies are very different, but this diversity of studied topics raises the quality of this special edition. Each of these studies represents an important stream of work. The first examines the influence of lifestyle on depression and health and indicates that watching television is the least risky type of screen behavior (Kidokoro et al.). This means that social media, online games, and online videos are more associated with a higher prevalence of depression. The following studies indicate: that proteinuria can be a predictor of frailty (Chang et al.); that physically active male participants tend to discriminate against those they consider overweight (Xu et al.); that a campus football programs have a high injury risk and that injury prevention and management strategies should be improved (Liu H. et al.); and that messages that place an exceptional emphasis on the benefit or harm of a certain behavior are much more effective in influencing motivation than messages that are simply presented to the public without a special explanation (Wang et al.).

## Conclusion

This Research Topic with its 24 studies very, very broadly covered the area of physical activity and lifestyle sustainability. The included studies indicate that the physical activity of subjects of all age categories is still constantly decreasing, even though the benefit of physical activity has long been proven (4, 5). However, published studies recommend various activities that have a positive effect on health and specify the minimum threshold of volume and intensity that is necessary to cause transformation in a positive direction, so they are extremely significant for practice. Furthermore, published studies confirm the damage that the COVID-19 pandemic has caused to humanity in the last few years, i.e., point to the fact that an adequate physical activity program that would prevent the decline of physical activity and fitness has not been found. Based on this, the question arises whether it was possible to design an adequate exercise program that could be carried out while respecting the rules on social distance, and thus strengthen immunity, and create the first line of defense of the organism so that it could more easily resist the virus? Also, the studies published in this Research Topic have designed several protocols for measuring, monitoring, and evaluating certain parameters, which will be used for improving the current situation in the field. Furthermore, this Research Topic reminded us of the strong influence of sociodemographic parameters on physical activities and physical fitness, but this is an area in which sports and health sciences are powerless to make changes. In the end, it drew attention to the risky behavior of those community members who need to change their behavior, and also to strategies that must be applied to improve the current situation in the field.

## Author contributions

BM drafted the editorial. SP, JJ, and RM revised and approved the final version. All authors contributed to the article and approved the submitted version.
